# Human endogenous retroviruses sustain complex and cooperative regulation of gene-containing loci and unannotated megabase-sized regions

**DOI:** 10.1186/s12977-015-0161-9

**Published:** 2015-04-17

**Authors:** Martin Sokol, Karen Margrethe Jessen, Finn Skou Pedersen

**Affiliations:** Department of Molecular Biology and Genetics, Aarhus University, Aarhus, DK-8000 Denmark

**Keywords:** Endogenous retrovirus and endogenous retrovirus-like repeats (ERVs), Chromatin immunoprecipitation with sequencing (ChIP-seq), Transcription coregulation, Paired-end RNA-sequencing (RNA-seq), LTR12 ERV9 LTR repeat, Alu SINE repeat, Mammalian apparent LTR retrotransposon (MaLR), Chimeric and unannotated transcription

## Abstract

**Background:**

Evidence suggests that some human endogenous retroviruses and endogenous retrovirus-like repeats (here collectively ERVs) regulate the expression of neighboring genes in normal and disease states; e.g. the human globin locus is regulated by an ERV9 that coordinates long-range gene switching during hematopoiesis and activates also intergenic transcripts. While complex transcription regulation is associated with integration of certain exogenous retroviruses, comparable regulation sustained by ERVs is less understood.

**Findings:**

We analyzed ERV transcription using ERV9 consensus sequences and publically available RNA-sequencing, chromatin immunoprecipitation with sequencing (ChIP-seq) and cap analysis gene expression (CAGE) data from ENCODE. We discovered previously undescribed and advanced transcription regulation mechanisms in several human reference cell lines. We show that regulation by ERVs involves long-ranging activations including complex RNA splicing patterns, and transcription of large unannotated regions ranging in size from several hundred kb to around 1 Mb. Moreover, regulation was found to be cooperatively sustained in some loci by multiple ERVs and also non-LTR repeats.

**Conclusion:**

Our analyses show that endogenous retroviruses sustain advanced transcription regulation in human cell lines, which shows similarities to complex insertional mutagenesis effects exerted by exogenous retroviruses. By exposing previously undescribed regulation effects, this study should prove useful for understanding fundamental transcription mechanisms resulting from evolutionary acquisition of retroviral sequence in the human genome.

**Electronic supplementary material:**

The online version of this article (doi:10.1186/s12977-015-0161-9) contains supplementary material, which is available to authorized users.

## Findings

In mammalian genomes retroviral elements have been adapted to fulfil essential biological functions as epitomized by the domestication of envelope fusion proteins, the syncytins, which mediate fusion of trophoblasts during placenta formation. Such diverse elements which are generally divided into human endogenous retroviruses and endogenous retrovirus-like elements (here collectively ERVs) comprise ~8% of the human genome and originate from cumulative germ line infections and retrotranspositions in our ancestors (reviewed in [1,2]). While the genomes of some mammals such as mouse and pig contain many active ERVs that may show high sequence similarity to their exogenous counterparts [3,4], there is no reported proof so far of recent ERV infection in humans. The most recently acquired HERV-K (HML2) family is considered potentially infectious, however, as functional viral proteins are encoded that produce non-infectious particles in teratocarcinomas and melanomas. Moreover, transcription of the HERV-K (HML2) consensus sequence results in the assembly of infectious particles that are inhibited by restriction factors including APOBEC family members [5,6].

While most ERVs are disrupted by fragmentation and mutations in the retroviral genes, the long terminal repeats (LTRs) preserve their function as either promoters or enhancers that may regulate adjacent human genes. In the human globin locus, an ERV9 modulates long-range transcription factor occupancies at several *cis*-linked genes that coordinate gene switching during normal hematopoiesis. The ERV9 also activates intergenic RNAs at low levels as a result of transient DNA looping with multiple intergenic sites at the globin locus [7]. In Hodgkin’s lymphoma aberrant activation of an LTR belonging to the THE1B subfamily of mammalian apparent LTR retrotransposons (MaLRs) promotes transcriptional activation of colony-stimulating factor 1 receptor (CSF1R) which is essential for tumor survival [8]. LTR-mediated activation is also associated with regulation of *TP63* (p63), a member of the tumor suppressor *TP53* (p53) family. In testis of Hominidae an ERV9 LTR functions as a strong promoter affecting novel isoform expression of *TP63* [9]. Similarly, cancer-specific isoform expression of the fatty acid binding protein 7 (*FAB7*) gene that is normally active in brain, is sustained by an LTR (LTR2-FABP) in diffuse large B-cell lymphoma [10].

ERV9 family members belong to the human ERV-I class and were repeatedly amplified during primate evolution [11]. The ERV9 family is estimated to comprise more than 120 loci and 4,000 copies of single LTRs, and therefore members of this family are distributed genome-widely and present on most chromosomes [12,13]. While exogenous retroviral insertional mutagenesis is commonly associated with complex host sequence activation [14-16], comparable transcription regulation by ERVs is less described. In this study, we exploited publically available deep sequencing data from ENCODE and previously established ERV9 consensus sequences [11] to identify transcription regulations sustained by members of this family, as well as other ERVs and/or repeat elements. For simplicity the term *chimeric* is here used to denote transcription covering ERV and adjacent sequence.

### RNA-sequencing exposes actively transcribed chimeric positions of ERV9 and ERV9-like repeats (collectively LTR12s)

**Figure 1:**
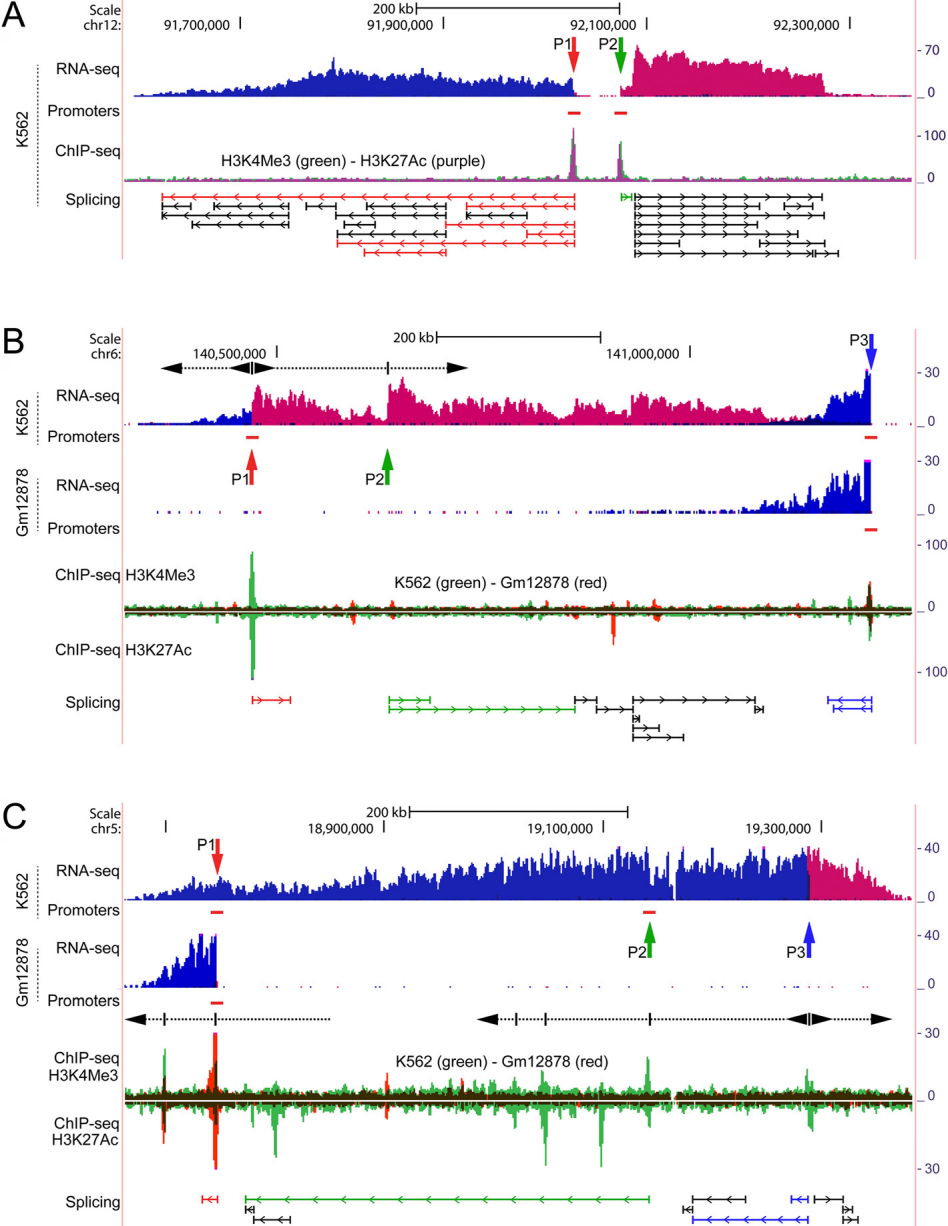
ERVs sustain complex and cooperative regulation of megabase-sized regions that are not annotated. RNA-seq coverages are shown as strand-specific BedGraphs [19]. Alignment to the positive and negative strands is colored red and blue, respectively. Horizontal red bars below the RNA-seq panels indicate positions of active promoters (not to scale), predicted from ChIP-seq by hidden Markov model (ENCODE/Broad). ChIP-seq peaks (ENCODE/Regulation) are colored according to subfigure legends. Right-hand axes show vertical viewing ranges of RNA-seq and ChIP-seq data. Vertical arrows, P1, P2 etc., indicate positions of ERVs and other repeats, and are colored according to linked (i.e. chimeric) RNA splice junctions of individual repeats. Splice junctions are from ENCODE/CSHL and ENCODE/Caltech. For simplicity only an excerpt is shown. Junctions in black are not linked to repeats in positions marked by vertical arrows. **(A-C)** Subfigures show cooperative transcription regulations of progressively increasing complexity in unannotated loci. **(A)** The ~700 kb locus is transcribed bidirectionally from separated sites, P1 and P2, containing ERV9-LTR12 and Alu repeats, respectively, and whose positions coincide with major ChIP-seq regulatory motifs representing promoters. **(B)** Transcription of the ~950 kb locus is sustained by ERVs and other repeats in positions P1-P3, including splicing from an Alu element positioned in P2. The position of P2 does not coincide with major ChIP-seq regulatory motifs in K562 cells, therefore the increase in transcription coverage at this position and chimeric splicing suggest contribution by an unknown mechanism. **(C)** The ~750 kb locus is regulated by three ERV9-LTR12s positioned in P1-P3. ChIP-seq enrichments at multiple positions suggest that coregulation by ERVs and non-LTR repeats may be more pervasive in some regions. In **(B-C)** the loci are differentially regulated in K562 and Gm12878 cells. Some hypothetical transcription patches are indicated (dashed arrows), based on increases in RNA-seq coverage and ChIP-seq enrichments. Close-ups in Figure 2.

**Figure 2:**
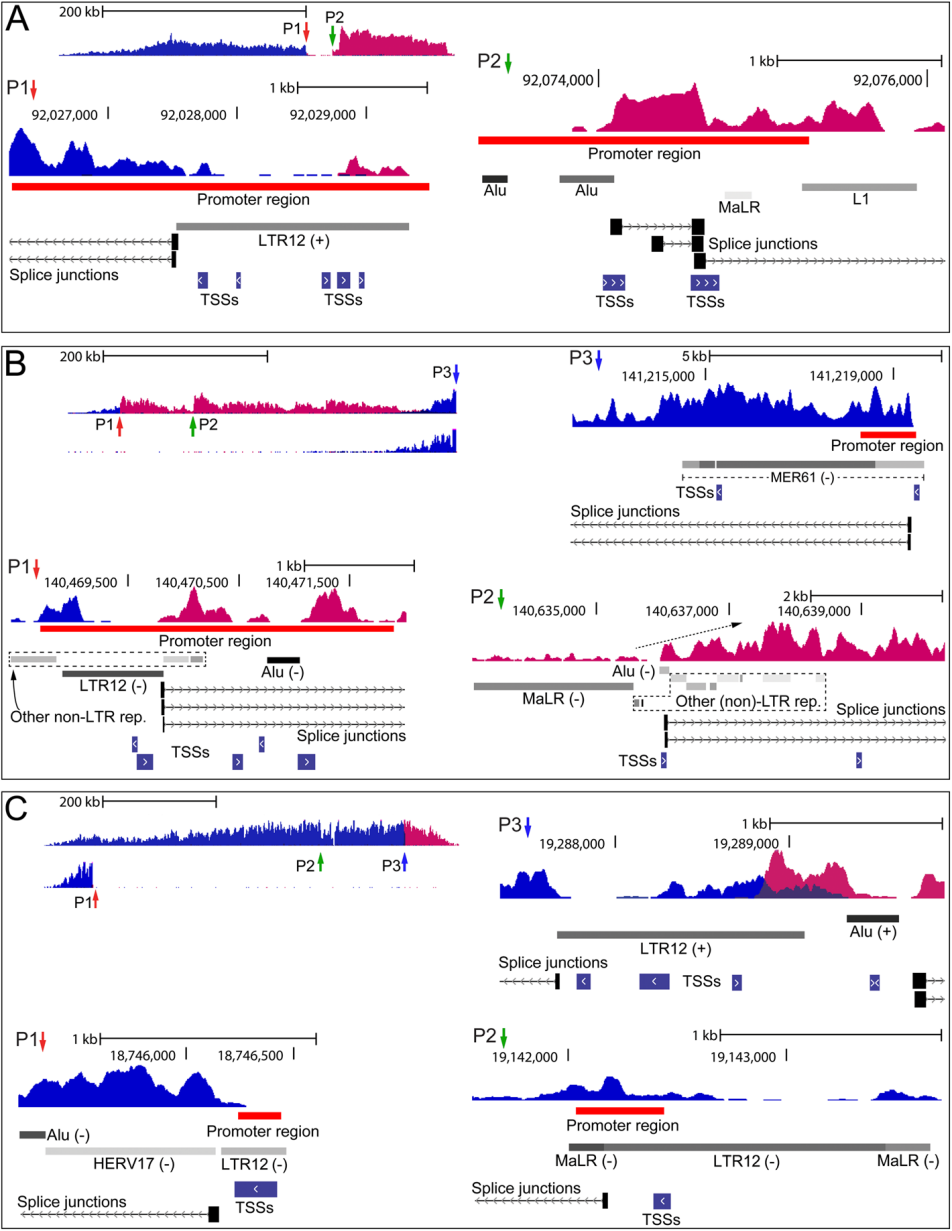
Close-up views of regulatory regions in unannotated loci. The subfigures **(A-C)** show close-up views of the positions P1, P2 and so on, shown in Figure 1. The LTR and non-LTR repeat elements are from RepeatMasker and shadings reflect the confidence of annotation where darker is higher [21]. CAGE TSSs are from ENCODE/Riken. The orientation (+ or -) of select repeats is shown with respect to the positive genome strand, and for simplicity some elements are not shown in the subfigures. The promoter regions are drawn to scale and correspond to those shown in Figure 1 from ENCODE/Broad. Only an excerpt of splice junctions is shown. In subfigure B, P2 the increase in RNA-seq coverage, immediately downstream of the MaLR repeat, is indicated by a dashed arrow. The subfigures show close-up views of regulatory regions in K562 cells, except for B, P3 and C, P1 which show corresponding regions from Gm12878 cells.

**Figure 3:**
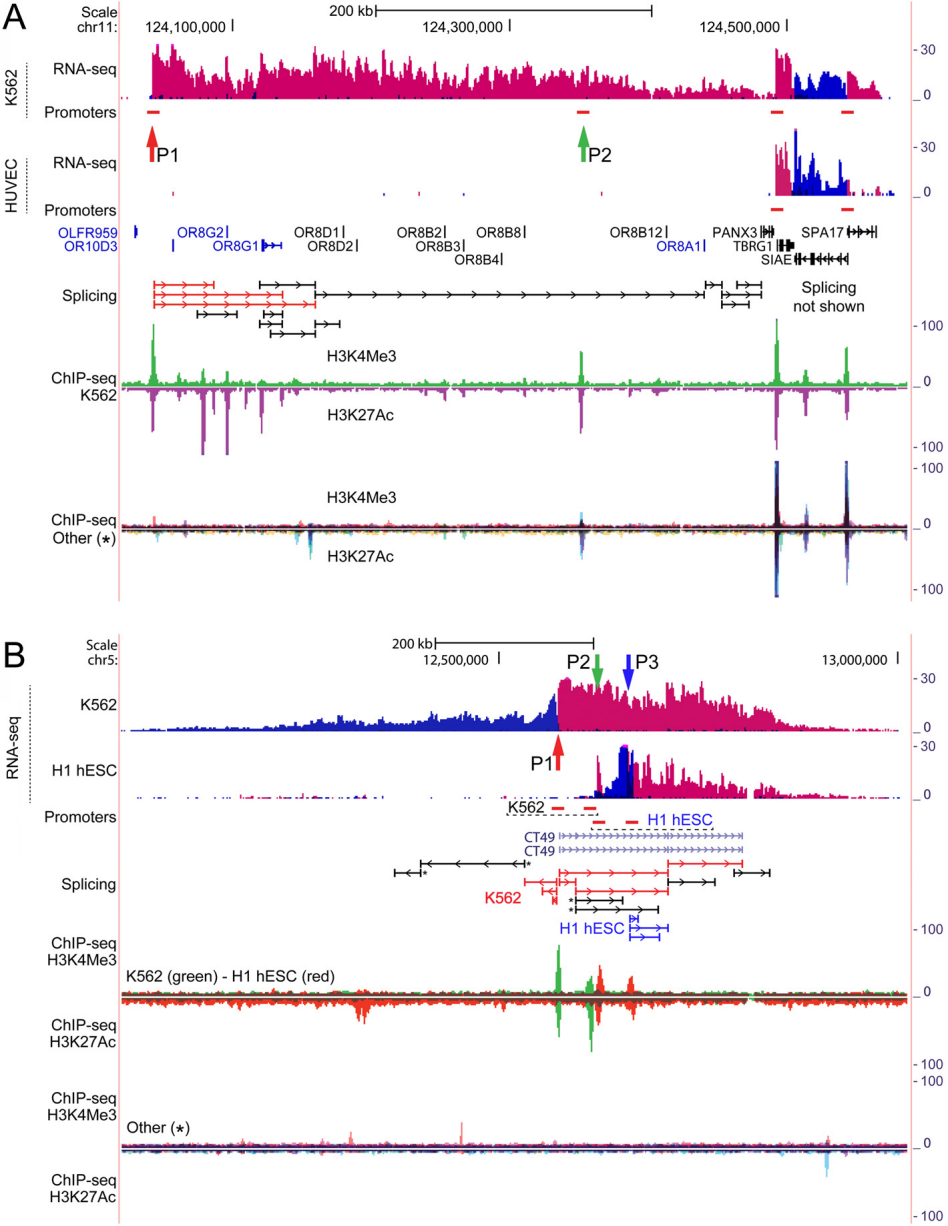
Complex regulation by ERVs is applicable also to annotated loci. **(A)** ERV9-LTR12 regulation of multiple olfactory gene-containing locus. Chimeric transcription from an ERV9-LTR repeat (P1, red vertical arrow) is associated with major activation of the olfactory locus, including (chimeric) splice junctions that span large distances in the locus. The RNA-seq coverage suggests that olfactory genes are activated strand-specifically (genes marked in blue are located on the positive strand) and include splicing into OR8G1. ChIP-seq shows that the position of the ERV9-LTR12 repeat coincides with a major H3K4Me3-H3K27Ac promoter marker in K562 cells. The role of a MIR in P2 is described in the main text. *Overlay view of ChIP-seq from Gm12878, H1 hESC, HSMM, HUVEC, NHEK and NHLF cell lines from ENCODE/Regulation. **(B)** Concurrent annotated and unannotated transcription of the CT49 locus. RNA-seq chimeric splice junctions show differential isoform expression of CT49 in H1 hESC (blue splice junctions) and K562 (red splice junctions). Transcription of CT49 is associated with unannotated transcription in the opposite direction in both cell lines. The positions P1-P3 coincide with ChIP-seq regulatory motifs in K562 (P1 and P2) and H1 hESC (P2 and P3), exclusively, showing that repeats present at these positions are major regulators of the CT49 locus (close-up views are shown in Figure 4). *Overlay view of ChIP-seq from Gm12878, HSMM, HUVEC, NHEK and NHLF cell lines from ENCODE/Regulation. Note that in this subfigure, P2 (green vertical arrow) marks two adjacently positioned promoter regions (horizontal red bars, not drawn to scale), i.e. one from both cell lines. Non-chimeric splice junctions marked with a star (*) are from K562, while the remaining black splice junctions are from either cell line. Please refer to Figure 1 legend for detailed description of data presentation.

**Figure 4:**
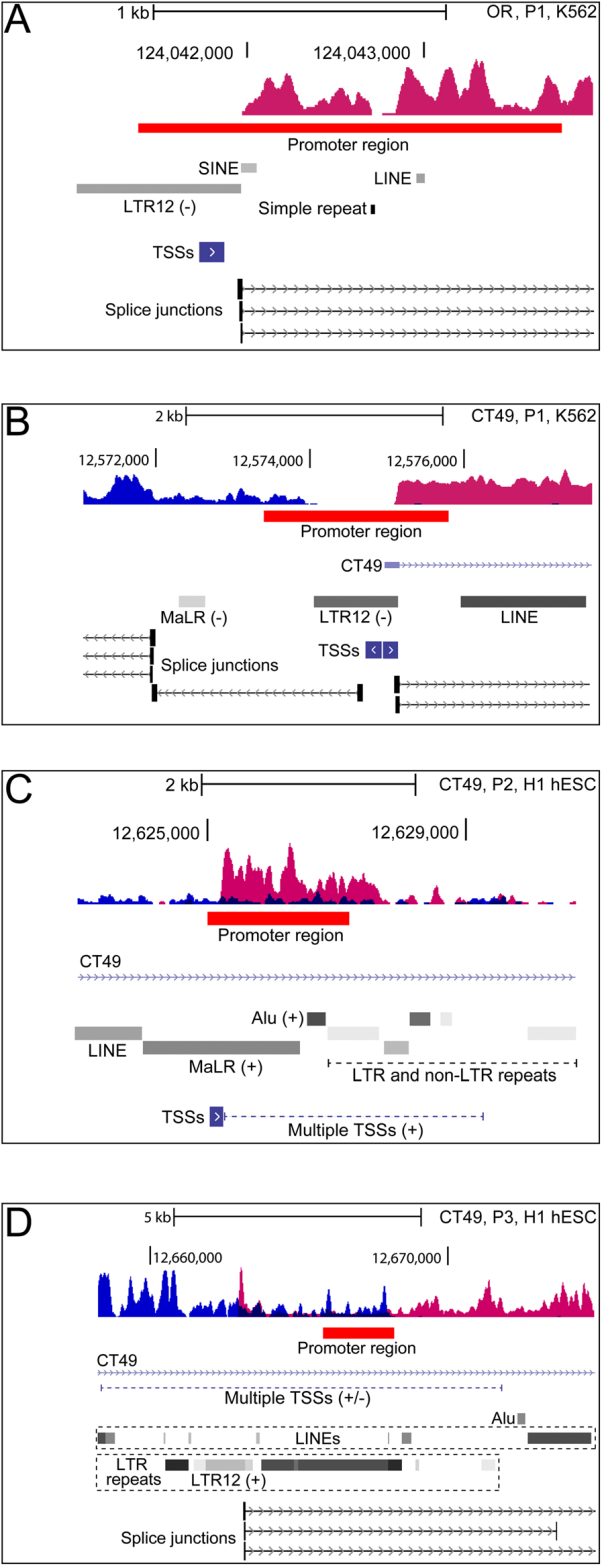
Close-up views of regulatory regions in the olfactory and CT49 loci. The subfigures show close-up views of regulatory positions P1, P2 and so on, from Figure 3. **(A)** In K562, the promoter position P1 corresponds to an ERV9-LTR12 repeat in the olfactory (OR) locus. **(B)** In K562, an ERV9-LTR is bidirectionally active giving rise to spliced transcript of opposite directions in the CT49 locus. **(C-D)** In H1 hESC the promoter positions (P2-P3) are positioned in repeat-dense regions in CT49 that contain multiple LTRs and non-LTR repeats, as well as multiple potential TSSs (directions indicated by + and –). The promoter regions are drawn to scale and correspond to those shown in Figure 3 from ENCODE/Broad. Please refer to the legend of Figure 2 details of data presentation.

We identified chimeric transcription in available non polyadenylation-selected directional long paired-end RNA-sequencing (RNA-seq) data from ENCODE/Cold Spring Harbor Lab (CSHL) [17] (Table 1), using a previously established approach [14]. In brief, 30 bp forward and reverse reads were aligned consecutively using Bowtie [18] against LTR U3 consensus sequences of 14 ERV9 families including also a joint consensus for all families [11]. We then filtered read pairs in which only one of the mates aligned to either of the consensus sequences. The corresponding unaligned mates were mapped in full length against the GRCh37/hg19 assembly of the human genome using TopHat [18] to include RNA splice junctions. The alignments were merged using BEDTools [19] in bins of 2 kb, and chimeric positions were assigned for each cell line based on at least 40 bp of uniquely mapped sequence located adjacently to ERV9s, ERV9-like repeats and other ERV1-type repeats that are represented broadly by more than 5,000 RepeatMasker LTR12 annotations in the human genome assembly [20-22]. Therefore, the chimeric positions (provided in Additional file 1) are supported by previously established consensus sequences as well as common repeat annotations. The transcription profiles shown in Figures 1, 2, 3 and 4 (described below) result from Bowtie/TopHat [18] mapping of full length forward reads followed by the conversion of unique alignments to BedGraphs using BEDTools [19].

**Table 1:** **RNA-sequencing data and mapping statistics**

**ENCODE/CSHL datasets**	**Positions**	**SF**
wgEncodeCshlLongRnaSeqHelas3CellLongnonpolyaFastqRd1Rep2.fastq	392	2.11
wgEncodeCshlLongRnaSeqHelas3CellLongnonpolyaFastqRd2Rep2.fastq		
wgEncodeCshlLongRnaSeqHepg2CellLongnonpolyaFastqRd1Rep1.fastq	295	1.26
wgEncodeCshlLongRnaSeqHepg2CellLongnonpolyaFastqRd2Rep1.fastq		
wgEncodeCshlLongRnaSeqK562CellLongnonpolyaFastqRd1Rep1.fastq	294	2.17
wgEncodeCshlLongRnaSeqK562CellLongnonpolyaFastqRd2Rep1.fastq		
wgEncodeCshlLongRnaSeqMcf7CellLongnonpolyaFastqRd1Rep1.fastq	140	0.76
wgEncodeCshlLongRnaSeqMcf7CellLongnonpolyaFastqRd2Rep1.fastq		
wgEncodeCshlLongRnaSeqGm12878CellLongnonpolyaFastqRd1Rep1.fastq	416	1.44
wgEncodeCshlLongRnaSeqGm12878CellLongnonpolyaFastqRd2Rep1.fastq		
wgEncodeCshlLongRnaSeqHuvecCellLongnonpolyaFastqRd1Rep1.fastq	267	1.40
wgEncodeCshlLongRnaSeqHuvecellLongnonpolyaFastqRd2Rep1.fastq		
wgEncodeCshlLongRnaSeqH1hescCellLongnonpolyaFastqRd1Rep1.fastq	355	1.91
wgEncodeCshlLongRnaSeqH1hescCellLongnonpolyaFastqRd2Rep1.fastq		
wgEncodeCshlLongRnaSeqHsmmCellLongnonpolyaFastqRd1Rep1.fastq	16	0.49
wgEncodeCshlLongRnaSeqHsmmCellLongnonpolyaFastqRd2Rep1.fastq		
wgEncodeCshlLongRnaSeqHmecCellLongnonpolyaFastqRd1Rep1.fastq	5	0.24
wgEncodeCshlLongRnaSeqHmecCellLongnonpolyaFastqRd2Rep1.fastq		
wgEncodeCshlLongRnaSeqNhekCellLongnonpolyaFastqRd1Rep1.fastq	202	1.53
wgEncodeCshlLongRnaSeqNhekCellLongnonpolyaFastqRd2Rep1.fastq		
wgEncodeCshlLongRnaSeqNhlfCellLongnonpolyaFastqRd1Rep1.fastq	18	0.39
wgEncodeCshlLongRnaSeqNhlfCellLongnonpolyaFastqRd2Rep1.fastq		

The table lists paired-end RNA-seq datasets from ENCODE/CSHL that were used to map positions of chimeric transcription. The scale factor (SF) which indicates the relative sequencing depth of each library was computed using Cufflinks [55].

We found that the number of positions varied across cell lines from only 5 to several hundred (Table 1), and although chimeric transcription was found in many cases to bring about comparable activation, numerous loci showed inconsistent activation, if any (Additional file 1, and data not shown). While this indicates that ERVs are differentially active, due to e.g. epigenetic silencing in some cell lines [23,24], the number of chimeric positions that can be mapped depends also on the depths of RNA-seq libraries. We found that the sequencing depth correlated positively with the number of chimeric positions (*R* = 0.88 and *p*-value = 3.29e–4, Pearson’s correlation, *N* = 11) (Table 1), suggesting that chimeric transcription may escape detection in some cell lines due to lower sequencing coverage [14].

In the following section complex transcription regulations are described based on integrative analyses of RNA-seq, chromatin immunoprecipitation with sequencing (ChIP-seq) and cap analysis gene expression (CAGE) data from ENCODE projects, as follows: ENCODE/CSHL (Table 1) and ENCODE/Caltech RNA-seq projects; annotation of active promoters from ChIP-seq by hidden Markov model (ENCODE/Broad); CAGE clusters of transcription start sites (TSSs) (ENCODE/Riken); ChIP-seq histone enrichments (ENCODE/Regulation) of H3K4Me3 and H3K27Ac that mark active regulatory motifs including promoters (H3K4Me3 or H3K4Me3/H3K27Ac) and enhancers (H3K27Ac and H3K4Me3 depletion) [25-31]. The particular use of each dataset is described in detail below. The projects are publically accessible through the UCSC genome browser [17] where the regulation patterns in Figures 1, 2, 3 and 4 (shown below) can be browsed.

### ERVs sustain complex and pervasive transcription regulation of large unannotated and gene-containing loci

We observed transcriptional regulation of genomic loci spanning from several hundred kilobases to around one megabase that do not contain gene annotations in common databases including UCSC. Unannotated transcription was found to proceed in both directions, including transcription of opposite genome strands, and initiating either bidirectionally, from a single position, and/or from distinct positions containing ERVs and other repeats (Figure 1 and close-up views in Figure 2, positions indicated by arrows P1, P2 etc.). The regulation patterns shared among cell lines are listed in Additional file 2: Figures S1.

In Figure 1A bidirectional transcription is separated into two large and interspaced patches of oppositely transcribed sequence in K562 cells, and is cooperatively sustained by an ERV9-LTR12 and densely positioned Alu repeats and an ERVL-MaLR family member among others (compare Figure 2A, P1-P2). Locally, at the position of the ERV9-LTR12, the RNA-seq coverage and clustering of TSSs indicate that this LTR is bidirectionally active (Figure 2A, P1), thereby sustaining sense and antisense transcription as described for other LTRs [16,32]. The positions of the LTR and non-LTR repeats coincide with regulatory motifs, as shown by strong coenrichment of ChIP-seq H3K4Me3-H3K27Ac promoter markers (Figure 1A). We found that among seven cell lines from ENCODE/Regulation strong coenrichment of H3K4Me3-H3K27Ac was exclusive for K562 cells, where the locus was also exclusively activated (Additional file 2: Figure S2). This suggests that the ERV9-LTR and other repeats positioned in P1 and P2 (Figure 1A) comprise the major regulatory motifs in this locus. Unannotated transcription also produces highly complex RNA splicing patterns (Figure 1A) that appear almost random suggesting that multiple cryptic splice signals are invoked. While the role of spliced transcripts is not known, the occurrence of aberrant RNA splicing is consistent with unannotated transcription patterns previously detected in gammaretrovirus-induced lymphomas [14], and is also associated with pseudogene activation [33].

The loci depicted in Figures 1B and C reveal progressively increasing complexities of human sequence regulation by ERVs and other repeat elements, as well as differential regulation among cell lines. In Figure 1B bidirectional transcription is shown to arise in a promoter region containing ERV9-LTR12 and Alu repeats, in K562 cells only (Figure 2B, P1), while furthest downstream a MER61-family ERV provirus activates transcription of negative polarity from a promoter region marked in both K562 and Gm12878 cells (Figures 1B and 2B, P3). In the intermediate region, which contains adjacently positioned ERVL-MaLR and Alu repeats among others (Figure 2B, P2), transcription coverage increases immediately downstream of the MaLR (Figure 2B, P2, dashed arrow) suggesting that this LTR contributes to transcription in K562 cells by an unknown mechanism. The Alu repeat contributes to RNA splicing by connecting far downstream sequence (compare Figures 1B, P2 and 2B, P2). Alu repeats are known to form a source of novel exon structures by providing cryptic splice signals tissue-specifically [34,35]. The regulation shown in Figure 1B therefore reveals a complex interplay of LTR and Alu repeats in sustaining transcription of this major unannotated locus and associated spliced transcripts. Transcription of the locus shown in Figure 1C shows differential regulation in K562 and Gm12878 cells that is comparable to that shown in Figure 1B. Interestingly, the locus in Figure 1C was found to contain four ERV9-LTR12 repeats (for clarity only three are shown in Figures 1C and 2C) of which only one contributes to transcription in Gm12878 cells resulting in activation of only a sub-region of the large locus. In K562 cells, however, transcription indicates that regulation is sustained cooperatively by several ERVs, involving possibly also an Alu repeat (compare profiles of K562 and Gm12878 in Figures 1C and 2C). The finding that only sub-regions of the large loci are activated in Gm12878 cells (Figures 1B and C) suggests that activation in some cases may be broken down into multiple sub-regions of unidirectional transcription, sustained by different ERVs and other non-LTR repeats. This is substantiated by CAGE TSSs (Figures 2B and C) as well as ChIP-seq promoter motifs in Figures 1B and C. These figures also show sub-regions of bidirectional transcription polarity, and some putative unidirectional sub-regions based on local increases in transcription coverage and/or enrichment of ChIP-seq motifs. As implied above, Figures 1B and C show differential ChIP-seq enrichment of the cell lines thereby confirming the regulatory roles of repeats in P1-P3 in these figures.

We found that complex regulation applies also to annotated loci containing non-coding RNAs and multiple genes, and concurrent transcription of adjacent unannotated and annotated sequence was also observed (Figure 3). In MCF7 and K562 cells a large 450-kb locus was activated containing 12 olfactory genes that encode odorant G protein–coupled receptors [36] (Figure 3A, and Additional file 2: Figure S3). The RNA-seq coverage shows that transcription of positive polarity, and initiating at a promoter region containing an ERV9-LTR12 element (Figure 3A, P1 and Figure 4A), covers almost the entire region of olfactory receptor genes (except OLFR959), four of which are located on the DNA plus strand (annotations shown in blue in Figure 3A), and of which one is annotated as putative (OR10D3). RNA (chimeric) splicing was found to connect OR8G1 and OR8A1 sequence to the promoter region containing the ERV9-LTR12 element. The transcription profile therefore shows that olfactory genes are activated strand-specifically as transcription of seven olfactory genes, located on the DNA minus strand, was not detected (including non-putative olfactory genes). The promoter region P2 in Figure 3A was found to contain a MIR repeat positioned on the negative genome strand, however the contribution of this repeat is uncertain as no splice junctions or CAGE TSSs clusters were found in this repeat (data not shown). The comparison of ChIP-seq H3K4Me3-H3K27Ac enrichments of K562 and 6 other cell lines, in which the olfactory locus was not activated, attributes a role of ERV9-LTR12 as a major transcriptional regulator of the olfactory locus (Figure 3A), and this was confirmed also in MCF7 cells (Additional file 2: Figure S3).

Transcription of the non-coding cancer-testis antigen 49 (CT49) locus in H1 hESC and K562 cells (Figure 3B) shows that bidirectional transcription concurrently activates annotated and unannotated sequence of positive and negative polarities, respectively. The activations resemble those in Figures 1B and C as regulation is differentially imposed in K562 and H1 hESC cells (compare P1-P3 in Figure 3B, note that P2 points to separate ChIP-seq promoters in the two cell lines), and sustained by different ERVs and/or repeats as shown by ChIP-seq enrichments of distinct regulatory regions in these cell lines only (compare profiles of H1 hESC and K562 and those of 6 other cell lines in Figure 3B). In K562 cells the promoter region P2 was found to contain LINE and Alu repeats, however the contribution of these repeats to transcription is uncertain as neither splice junctions nor CAGE TSSs clusters were identified in the vicinity of P2 (data not shown). It therefore seems likely that regulation in K562 is sustained solely by an ERV9-LTR12 repeat in position P1 (Figure 4B). In H1 hESC cells on the other hand, the promoters (P2 and P3) are situated in repeat-dense positions containing multiple LTRs and non-LTR repeats, as well as multiple potential TSSs (Figures 4C and D). The assignment of separate regulatory roles to individual repeats is therefore not trivial. The RNA-seq coverage, however, suggests that transcription is sustained by LTR-repeats, and this is supported by RNA splice junctions in one of the sites (compare Figures 4C and D). While it was shown that an ERV9 drives isoform expression of *TP63* in testis of Hominidae [9] (described above), transcription of CT49 in K562 and H1 hESC cells is peculiar as regulation supposedly affects expression of cell-specific isoforms resulting in turn from differences in epigenetic regulation of LTR and non-LTR repeats in cell lines as shown by ChIP-seq.

### Discussion and conclusion

We have exposed regulatory patterns that attribute a pivotal role of ERVs in sustaining complex and pervasive transcription of the human genome, in some cases involving cooperative effects by several ERVs and non-LTR repeats. This was demonstrated by RNA-seq and CAGE that showed complete shifts of transcription polarity, and/or initiation of transcription at LTRs and non-LTR repeats (e.g. Figures 2A, P1 and 4, P1). We also detected recurrent association of repeats with ChIP-seq regulatory motifs, selectively in cell lines where transcription was activated (Figures 1 and 3, and Additional file 2: Figures S2-S3). Moreover, chimeric splicing was found to connect sequence of ERVs and non-LTR repeats over large distances in transcribed loci, suggesting that long-spanning and processed chimeric transcripts are produced.

Interestingly, in some loci recurrent association with ChIP-seq regulatory motifs implied that transcription is sustained by a limited number of major regulators, whereas the presence of multiple potential regulators in other loci showed that coregulation by LTRs and non-LTR repeats may be more pervasive (e.g. compare Figures 3A and B). The unannotated and gene-containing loci span from several hundred kilobases up to one megabase, thereby encompassing multiple repeat and non-repeat sequences that may possibly contribute to transcription. We found that in some loci regulation was cooperatively sustained by ERV9-LTR12 and other ERVs including also non-LTR repeats of the short interspersed elements (SINE) group such as Alu repeats, and long interspersed elements (LINEs) (e.g. Figures 1B and corresponding close-up views in Figure 2B). LINEs drive transcription through RNA polymerase II and may transpose autonomously [37]. Alu repeats comprise the highest copy-number of non-LTR retrotransposons in the human genome constituting more than one million repeats and their mobility is LINE-1 dependent [38,39]. Alus drive expression of noncoding RNAs through RNA polymerase III transcription [40], and are known to evolve as cellular enhancers indicating that a countless fraction of Alus form a reservoir of proto-enhancers in the human genome [41]. The advent of genomics has highlighted the complex architecture of eukaryotic promoters which can be considered a range of regulators that contain multiple sequence motifs making possible highly specific transcription regulation. The promoter regions may contain promoter-proximal enhancers and rely on coopted motif usage in case of promoters with diverged nucleotide compositions [42-45]. The attribution of separate functions to individual LTRs and non-LTR repeats in repeat-dense promoter regions (e.g. Figures 4C and D) may therefore appear redundant and suggests that locally, transcription may be cooperatively regulated in some loci. Under certain circumstances, Alus may provide cryptic splice signals resulting in aberrantly spliced RNA transcripts [35], and in this study splicing through Alu repeats was also detected (e.g. Figure 1A and corresponding close-up view in Figure 2A).

While bidirectional transcription separates activation into distinct patches or sub-regions of transcription in the forward and reverse directions, respectively (e.g. Figures 1A and 3B), long-reaching unidirectional transcription is more difficult to discern as indicated by the multiple hypothetical sub-regions in Figures 1B and C. We speculate if activation of potential sub-regions in some cases may result from higher-order chromatin interactions whereby major regulators are brought into proximity of potential repeat and/or non-repeat promoters. In support of the existence of putative sub-regions, enrichment of CTCF (CCCTC-binding factor)–bound positions at intermediate locations was observed in the loci shown in Figures 1 and 3 (data not shown), suggesting that genome architectural motifs possibly contribute to regulation [46]. It is widely established that enhancers may act over very large distances and interact with multiple promoters [25,47,48], and activation through this mechanism has been described for some LTRs as well as non-LTR repeats [40,49-51]. In fact, the human globin locus is regulated in this way by an ERV9 LTR whereby transcription initiates from other promoters in the globin locus [7] (described above). Therefore, activation of the globin locus suggests a hypothetical mode of regulation of e.g. the olfactory locus (Figure 3A), as well as the putative sub-regions in large unannotated loci (Figures 1B and C).

Transcription of unannotated regions encompassing several hundred kilobases to almost one megabase is of fundamental interest as the sheer amount of genome sequence and associated RNA splicing patterns suggest transcription of multiple unknown RNAs. We detected concurrent unannotated and annotated transcription at the CT49 locus of negative and positive polarity in both K562 and H1 hESC cells (Figure 3B). In K562, an ERV9-LTR12 induced transcription in both directions (Figure 4B) showing that the LTR is bidirectionally active, giving rise to spliced transcripts of opposite polarities. The promoters of many protein-coding genes are known to sustain transcription of non-coding RNAs in the opposite direction, and bidirectional transcription therefore appears to be an intrinsic feature of promoters [52,53], and this applies also to promoters of some endogenous and exogenous retroviral LTRs [16,32]. The concept of transcriptional bidirectionality is applied also to phenomena of intragenic and intronic transcription of overlapping genome strands [53] as observed in H1 hESC cells (Figure 4D), and this is also associated with non-coding RNA expression [54].

Coregulation of transcription by multiple endogenous LTRs and associated non-LTR repeats in large unannotated and gene-containing loci reveals a higher order complexity of human genome regulation that to our knowledge was not shown before. We have previously exposed transcription patterns of similar complexity sustained by exogenous retroviruses in mouse tumors using RNA-seq and ChIP-seq [14]. It should therefore be important to consider the extent to which human endogenized retroviruses sustain regulation in primary cells and tissues that is comparable to that observed in human reference cell lines. Considering the vast amount of retroviral sequence in the genome it is expected that regulation of equivalent or similar complexity is likely to occur genome-wide in normal and pathologically altered cells. Taken together, this study should provide a useful framework for understanding fundamental and complex transcription regulatory principles resulting from evolutionary acquisition of retroviral sequence in the human genome.

## Additional files

Additional file 1:
**List of ERV9-LTR12 chimeric positions identified in 11 human cell lines using RNA-seq.** The spreadsheet lists unfiltered chimeric positions mapped in cell lines from ENCODE/Cold Spring Harbor Lab [17] based on U3 consensus sequences of 14 ERV9 families and one joint consensus [11]. The alignments where merged in 2 kb bins, and therefore some positions may represent the same genomic locus due to separation of paired-end sequencing reads by RNA splicing. Additional file 2:
**(this file contains supplementary Figures S1-S3). Figure S1.** List of common regulation patterns in cell lines. The table lists cell lines for which the loci shown in Figures 1 and 3 were fully or partially activated. **Figure S2.** ChIP-seq attributes a major regulatory function of ERVs and other repeats in sustaining transcription of large unannotated loci. The locus corresponds to the one shown in Figure 1A and was activated in K562 cells only. Comparison of ChIP-seq enrichments show that the positions of ERV9-LTR12 and Alu repeats in positions P1 and P2, respectively, coincide with major histone H3K4Me3-H3K27Ac coenrichments in K562, exclusively. (*) Overlay view of ChIP-seq peaks from Gm12878, H1 hESC, HSMM, HUVEC, NHEK and NHLF cell lines from ENCODE/Regulation. In the bottom of the subfigure RNA-seq coverage from ENCODE/CSHL of the seven cell lines is shown in dense view, and is separated into coverage on the minus (−) and plus (+) genome strands. Coloring of cell line names corresponds to coloring of ChIP-seq peaks. **Figure S3.** An ERV9-LTR12 is a major regulator of the olfactory locus in K562 and MCF7 cells. The figure shows RNA-seq coverages from K562 and MCF7 cells across the olfactory locus, as well as ChIP-seq promoter hotspots (Ht) from the ENCODE/University of Washington project. The black arrow marks the position of an ERV9-LTR12 (P1) that coincides with a promoter hotspot present exclusively in MCF7 and K562 cells where the olfactory locus was activated. The coloring of cell line names in this figure is arbitrary.

## Abbreviations

LTR: Long terminal repeat
RNA-seq: RNA sequencing
ERVs: Endogenous retroviruses and endogenous retrovirus-like elements
MaLRs: Mammalian apparent LTR retrotransposons
ChIP-seq: Chromatin immunoprecipitation with sequencing
CAGE: Cap analysis gene expression
TSSs: Transcription start sites
SINEs: Short interspersed elements
LINEs: Long interspersed elements
