# Supplementary material for: Human endogenous retroviruses sustain complex and cooperative regulation of gene-containing loci and unannotated megabase-sized regions
Source: Retrovirology. 2015 Apr 17;12:32. doi: 10.1186/s12977-015-0161-9 (PMC4422309; doi:10.1186/s12977-015-0161-9)
Supplement: Additional file 2: — (this file contains supplementary Figures S1-S3). Figure S1. List of common regulation patterns in cell lines. The table lists cell lines for which the loci shown in Figures 1 and 3 were fully or partially activated. Figure S2. ChIP-seq attributes a major regulatory function of ERVs and other repeats in sustaining transcription of large unannotated loci. The locus corresponds to the one shown in Figure 1A and was activated in K562 cells only. Comparison of ChIP-seq enrichments show that the positions of ERV9-LTR12 and Alu repeats in positions P1 and P2, respectively, coincide with major histone H3K4Me3-H3K27Ac coenrichments in K562, exclusively. (*) Overlay view of ChIP-seq peaks from Gm12878, H1 hESC, HSMM, HUVEC, NHEK and NHLF cell lines from ENCODE/Regulation. In the bottom of the subfigure RNA-seq coverage from ENCODE/CSHL of the seven cell lines is shown in dense view, and is separated into coverage on the minus (−) and plus (+) genome strands. Coloring of cell line names corresponds to coloring of ChIP-seq peaks. Figure S3. An ERV9-LTR12 is a major regulator of the olfactory locus in K562 and MCF7 cells. The figure shows RNA-seq coverages from K562 and MCF7 cells across the olfactory locus, as well as ChIP-seq promoter hotspots (Ht) from the ENCODE/University of Washington project. The black arrow marks the position of an ERV9-LTR12 (P1) that coincides with a promoter hotspot present exclusively in MCF7 and K562 cells where the olfactory locus was activated. The coloring of cell line names in this figure is arbitrary. [file 12977_2015_161_MOESM2_ESM.pptx]

## Slide 1
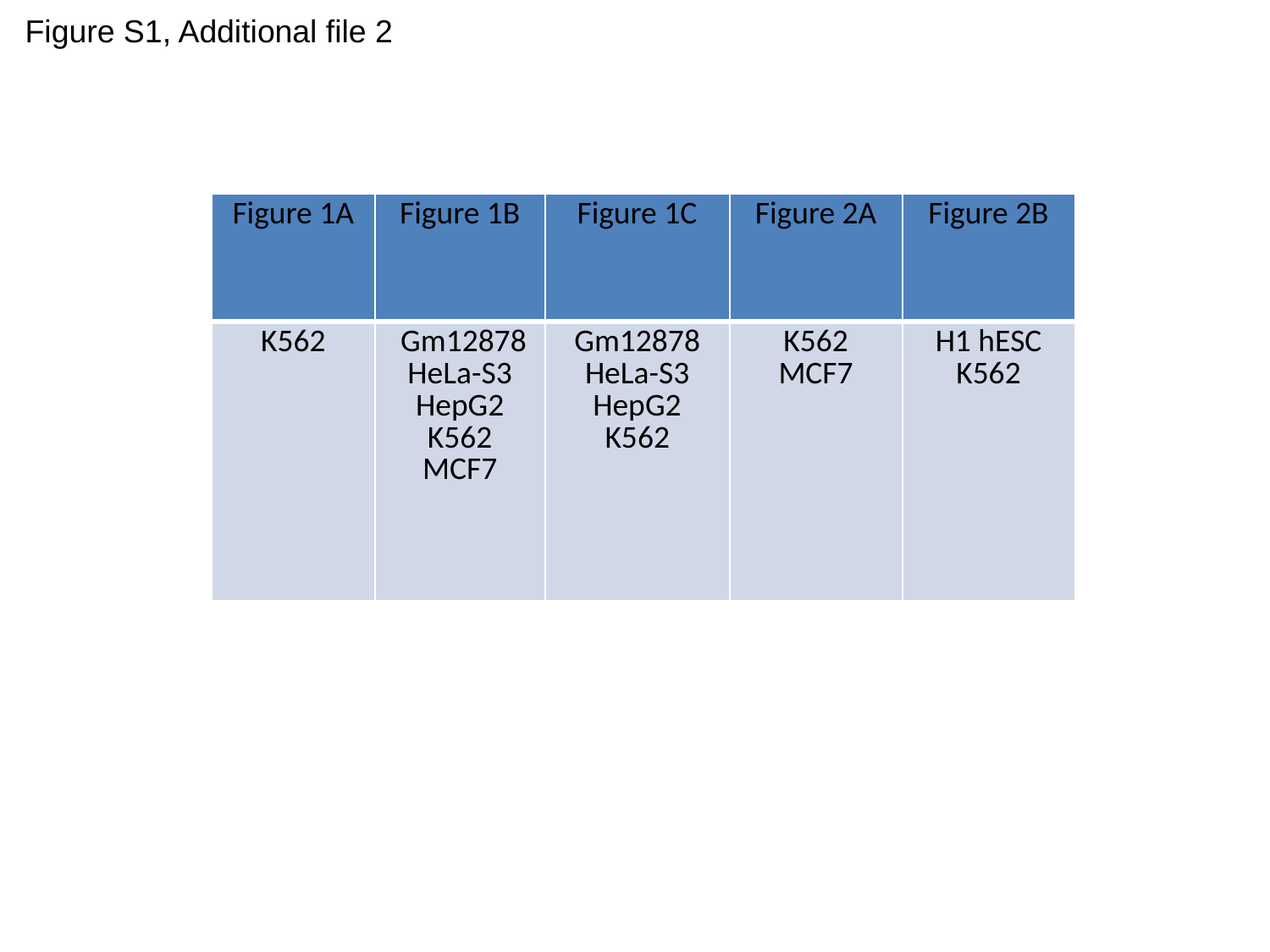

Figure S1, Additional file 2
| Figure 1A | Figure 1B | Figure 1C | Figure 2A | Figure 2B |
| --- | --- | --- | --- | --- |
| K562 | Gm12878 HeLa-S3 HepG2 K562 MCF7 | Gm12878 HeLa-S3 HepG2 K562 | K562 MCF7 | H1 hESC K562 |

## Slide 2
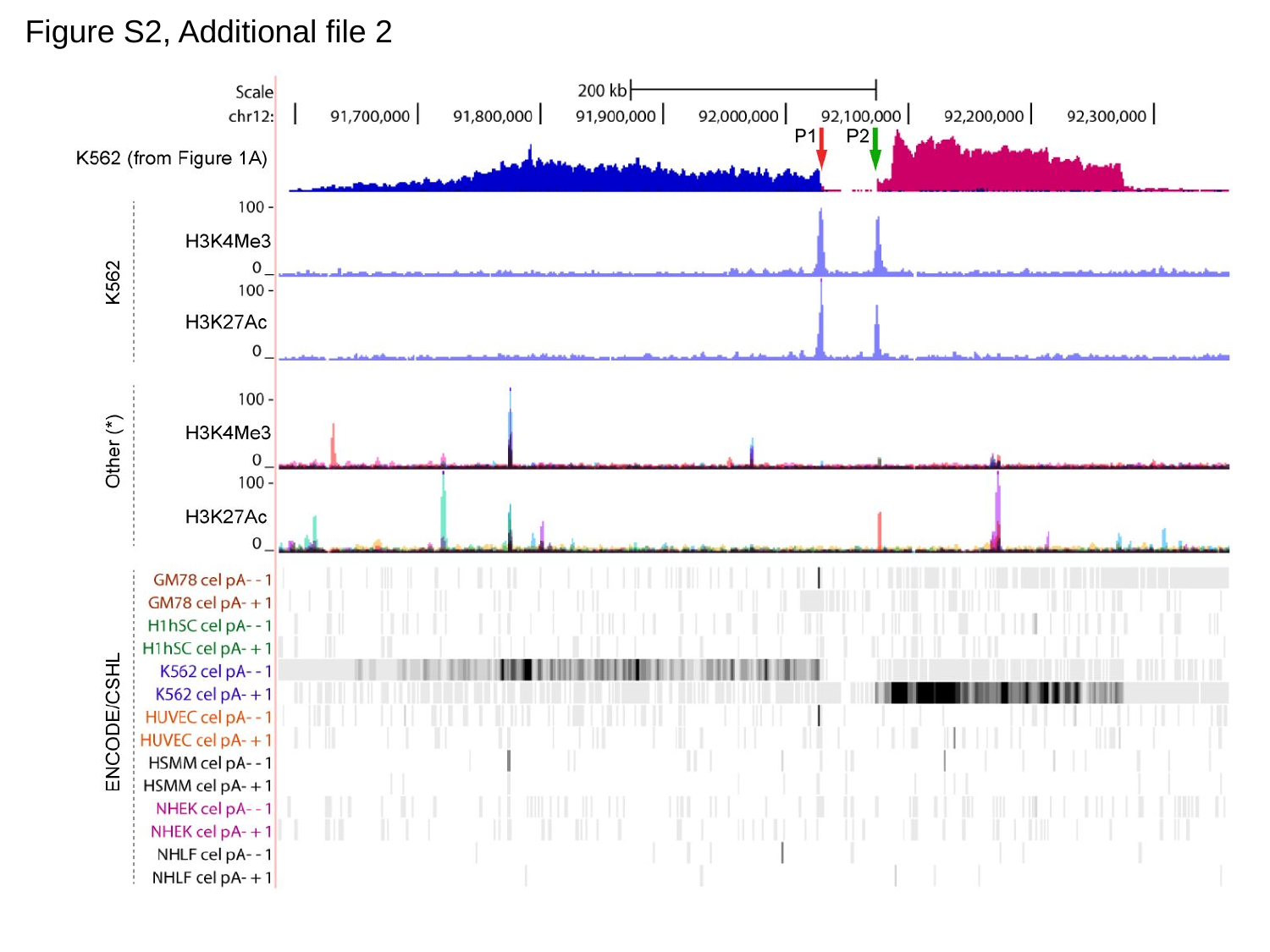

Figure S2, Additional file 2

## Slide 3
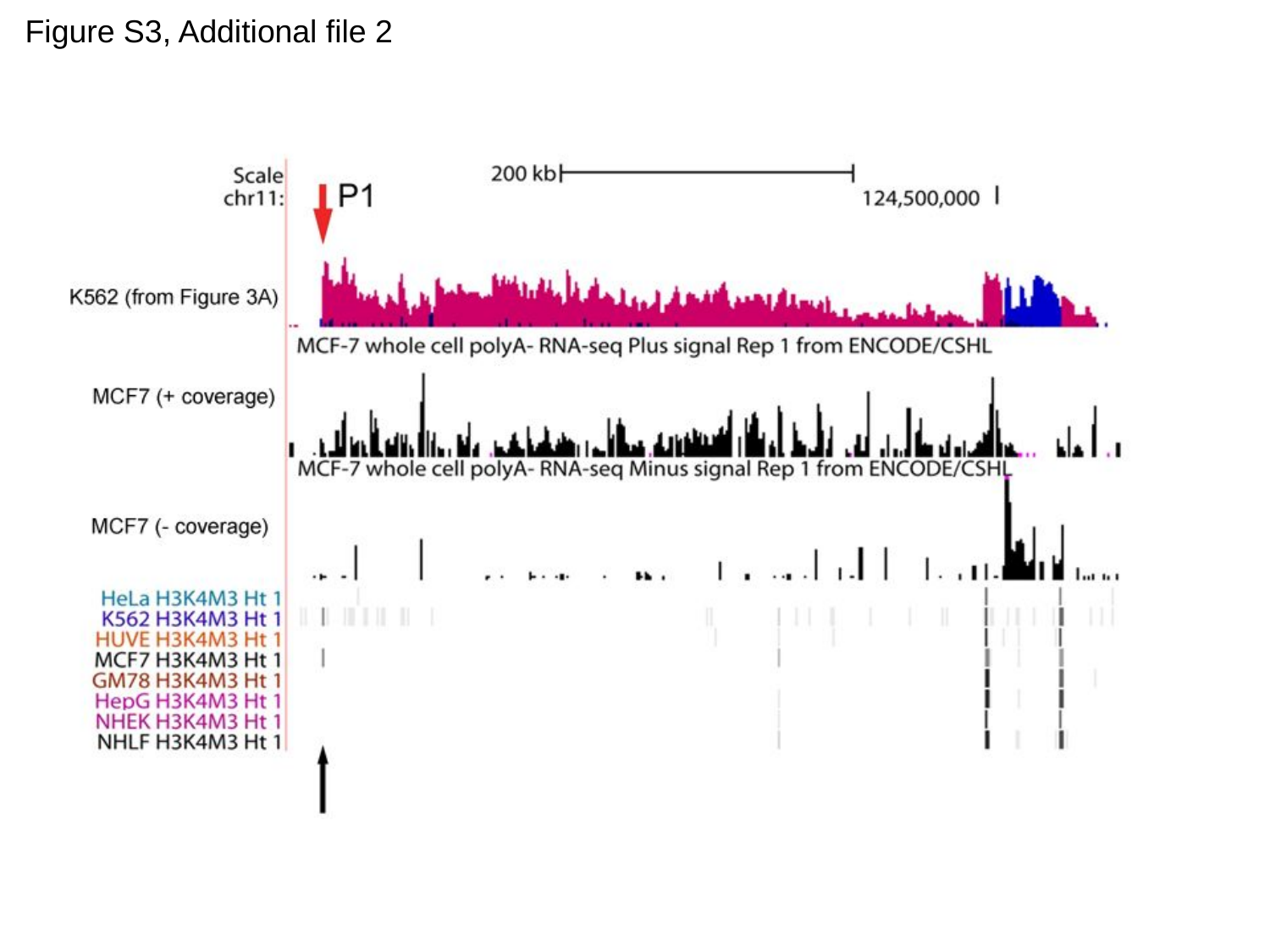

Figure S3, Additional file 2
